# Genome-Wide Scan Identifies Variant in *TNFSF13* Associated with Serum IgM in a Healthy Chinese Male Population

**DOI:** 10.1371/journal.pone.0047990

**Published:** 2012-10-31

**Authors:** Ming Yang, Yongming Wu, Yanmei Lu, Changyuan Liu, Jielin Sun, Ming Liao, Min Qin, Linjian Mo, Yong Gao, Zheng Lu, Chunlei Wu, Youjie Zhang, Haiying Zhang, Xue Qin, Yanling Hu, Shijun Zhang, Jianling Li, Min Dong, S. Lilly Zheng, Jianfeng Xu, Xiaobo Yang, Aihua Tan, Zengnan Mo

**Affiliations:** 1 Center for Genomic and Personalized Medicine, The First Affiliated Hospital of Guangxi Medical University, Nanning, Guangxi, People's Republic of China; 2 Center for Cancer Genomics, Wake Forest University School of Medicine, Winston-Salem, North Carolina, United States of America; 3 Institute of Urology and Nephrology, the First Affiliated Hospital of Guangxi Medical University, Nanning, Guangxi, People's Republic of China; 4 Department of Occupational Health and Environmental Health, Guangxi Medical University, Nanning, Guangxi, People's Republic of China; 5 Department of Clinical Laboratory, the First Affiliated Hospital of Guangxi Medical University, Nanning, Guangxi, People's Republic of China; 6 Medical Scientific Research Center, Guangxi Medical University, Nanning, Guangxi, People's Republic of China; 7 Department of Pharmacology, Guangxi Medical University, Nanning, Guangxi, People's Republic of China; 8 Institute of Cardiovascular Disease, the First Affiliated Hospital of Guangxi Medical University, Nanning, Guangxi, People's Republic of China; 9 Center for Genetic Epidemiology, Van Andel Research Institute, Grand Rapids, Michigan, United States of America; 10 Fudan-VARI Center for Genetic Epidemiology, Fudan University, Shanghai, People's Republic of China; 11 Fudan University Institute of Urology, Huashan Hospital, Shanghai, People's Republic of China; 12 Department of Chemotherapy, the Affiliated Tumor Hospital of Guangxi Medical University, Nanning, Guangxi, People's Republic of China; The Children's Hospital of Philadelphia, United States of America

## Abstract

IgM provides a first line of defense during microbial infections. Serum IgM levels are detected routinely in clinical practice. And IgM is a genetically complex trait. We conducted a two-stage genome-wide association study (GWAS) to identify genetic variants affecting serum IgM levels in a Chinese population of 3495, including 1999 unrelated subjects in the first stage and 1496 independent individuals in the second stage. Our data show that a common single nucleotide polymorphism (SNP), rs11552708 located in the *TNFSF13* gene was significantly associated with IgM levels (*p* = 5.00×10^−7^ in first stage, *p* = 1.34×10^−3^ in second stage, and *p* = 4.22×10^−9^ when combined). Besides, smoking was identified to be associated with IgM levels in both stages (*P*<0.05), but there was no significant interaction between smoking and the identified SNP (*P*>0.05). It is suggested that *TNFSF13* may be a susceptibility gene affecting serum IgM levels in Chinese male population.

## Introduction

IgM is the first antibody to be produced during an immune response, the first to appear during ontogeny and is also the oldest, being the sole class of antibody to be present in all vertebrate species [1]. IgM plays an important role in the immunology of health and disease. Whereas the role of natural IgM as the first line of defense for protection against invading microbes has been extensively investigated, more recent reports have highlighted their potential roles in the maintenance of tissue homeostasis via clearance of apoptotic and altered cells through complement-dependent mechanisms, inhibition of inflammation, removal of misfolded proteins, and regulation of pathogenic autoreactive IgG antibodies (Abs) and auto-antibody-producing B cells [2].

IgM is produced by two different B-lymphocyte populations. B1-cells synthesize IgM called natural antibodies (NA), which is not connected with immunization [3]. B2-lymphocytes produce IgM as a reaction to antigenic stimulus [4]. Low levels of IgM might increase the risk of infection, as well as exacerbate autoimmunity and increase the risk of atherosclerosis [5].

Reports of pedigree studies or twin studies have shown that genetic factors are important in determining serum total immunoglobulin and specific antibody levels in human [6], with genetic heritability for IgM ranging from 45% to 55% [7]. Several studies in a population of common variable immunodeficiency (CVID) patients implied that there might be an association between some gene loci and serum IgM levels [8]. So far, comprehensive genetic assessments of the variability in serum IgM levels are limited. Besides, the allele and genotype frequencies, and linkage disequilibrium (LD) patterns differed across the populations. While, common genetic variants that influence serum IgM levels could be important for identifying persons at risk for IgM disorder and enhancing our understanding of the observed associations between serum IgM status and several diseases. In this study, we conducted a two-stage GWAS in a Chinese population in search of population-specific genetic variations associated with serum IgM levels.

## Materials and Methods

### Study participants

Stage 1 of the GWAS included 1999 unrelated healthy Chinese men age 20–69 years old from the Fangchenggang Area Male Health and Examination Survey (FAMHES). The FAMHES is described elsewhere [9]. Briefly, it was designed to investigate the effects of environmental and genetic factors and their interaction with the development of age-related chronic diseases. All men who participated in physical examinations in the Medical Centre of Fangchenggang First People's Hospital from September 2009 to December 2009 were invited to participate in the study (n = 4364). A total of 4303 participants (98.6%) consented and donated blood samples. The participants in stage 1 were randomly selected from these men who met age criteria. All participants self reported that they were of southern Chinese Han ethnicity.

Stage 2 of the GWAS consisted of 1496 healthy Chinese men age 20–69 years old. They were randomly selected from male participants who participated in physical examinations from September 2009 to September 2010 in the Medical Centre of Fangchenggang First People's Hospital, Guigang People's Hospital and Yulin First People's Hospital. The stage 2 samples from Fangchenggang First People's Hospital were independently recruited from the stage 1 samples. Among these participants, 996 were of Han ethnicity and 500 were of Zhuang ethnicity.

The same recruitment strategy was used in stages 1 and 2. Comprehensive health information was collected through clinical examination, and additional demographic information was obtained via a standardized questionnaire. All participants self-reported to be free of diabetes mellitus, coronary heart disease, stroke, hyperthyroidism, rheumatoid arthritis, tumors, Systemic lupus erythematosus (SLE), Celiac disease and impaired hepatic or renal function. We obtained written documentation of informed consent from all study participants, and the research protocol was approved by the local Ethics Committee. Drinking behavior was assessed on the basis of a self-administered life-style questionnaire. Alcohol consumption was classified into two categories: drinkers and non-drinkers. Respondents that reported drinking any beverage more often than ‘less than once a year’ or ‘never’ were coded as drinkers [9], [10].

### Measurement of IgM

The description of the laboratory test has been previously reported in detail [11]. Briefly, overnight fasting venous blood specimens were collected between 8:00AM and 11:00AM, and were transported frozen to the testing center of Department of Clinical Laboratory at the First Affiliated Hospital of Guangxi Medical University in Nanning in two hours, which were centrifuged within 15 to 25 minutes and stored at −80°C until analysis. IgM was measured with electrochemiluminescence immunoassay on COBAS 6000 system E601 (Elecsys module) immunoassay analyzer (Roche Diagnostics, GmbH, Mannheim, Germany) with the same batch of reagents, and the inter-assay coefficient of variation was 4.97%.

### SNP genotyping

Two different platforms were used for SNP genotyping. The Illumina Omni 1 platform was used for a genome-wide assay of samples in stage 1. The Sequenom iPLEX system (Sequenom, Inc., San Diego, CA, USA) was used in the second stage. Polymerase chain reaction and extension primers were designed using Mass ARRAY Assay Design 3.1 software (Sequenom, Inc.). Genotyping procedures were performed according to the manufacturer's iPLEX Application Guide (Sequenom Inc.). All genotyping reactions were performed in 384-well plates. Each plate included a duplicate for three or four participants selected at random, as well as six to nine negative controls in which water was substituted for DNA. The average concordance rate was 99.8%.

### Statistical analysis

First-degree cryptic relationships were evaluated via an identity-by-descent (IBD) analysis. Then, quality control (QC) procedures were applied to 1999 unrelated individuals that were genotyped using the Illumina Omni-Express platform. A total of 1999 individuals passed the call rate of 95% and were used in the final statistical analysis. We then applied the following QC criteria to filter SNPs: *P*<0.001 for the Hardy–Weinberg equilibrium test, minor allele frequency <0.01 and genotype call rate <95%. Based on these criteria, 709 211 SNPs were retained. The IMPUTE computer program [12] was then used to infer the genotypes of SNPs (e.g. SNPs catalogued in Hapmap Phase II CHB population release #24) in the genome that was not directly genotyped. A posterior probability of >0.90 was applied to call genotypes that were imputed using IMPUTE software. After applying the same QC criteria, as used above, a total of 1 940 243 SNPs remained in the final analysis.

IgM values showed a markedly skewed distribution, and natural logarithmic (ln) transformations were performed to approximate normality (Figure S1, S2). Analysis for IgM was performed on log-transformed values. SNP association tests were performed using linear regression implemented in PLINK [13] under the assumption of an additive relationship between the number of copies of the minor allele. Population stratification was estimated by a principal component approach, as implemented by EIGENSTRAT software [14]. The top two Eigens were adjusted as covariates in the linear regression analysis. Clinical covariates utilized in the linear regression modeling included age at the time of IgM measurement, alcohol intake (yes, no), and cigarette smoking (yes, no).

For regions with multiple SNPs that were significant at *P*<10^−7^, multivariate linear regression analysis was applied to test the independence of the respective SNPs. Only the SNPs that remained significant at 10^−7^ in the multivariate analysis were selected. The combined analysis of two-stage data was performed using a linear regression, adjusting for the covariates and stage information. For the first stage, we have 80% power to detect a 0.155 of difference IgM (with a mean of 1.37, standard deviation of 0.68), based on 1,999 samples, assuming MAF 0.2 and *P*-value of 5E−07. For the second stage, we have 80% power to detect a 0.07 of difference IgM (with a mean of 1.26, standard deviation of 0.46), based on 1,496 samples, assuming MAF 0.2 and *P*-value of 1.25E−02.

## Results

The demographics of the individuals in this two-stage GWAS have been described in our prior study [11]. 1999 participants in stage 1 and 1496 participants in stage 2 were available for analysis. No significant difference was observed between the two stages in age distribution (37.54 vs 37.31 yrs, *p* = 0.54), body mass index (BMI) (23.31 vs 23.46 kg.m^−2^, *p* = 0.18) and smoking behavior (*p* = 0.66), excepting for alcohol consumption (*p* = 0.02) (Table 1). There were significant differences on serum IgM levels between drinkers and non-drinkers in the first stage (*P* = 0.013), whereas no significant difference was detected in the second stage (*P* = 0.129). Besides, Smokers had significantly lower IgM levels than non-smokers in both stages (Table S1).

**Table 1:** General characteristics of the two-stage GWAS study participants.

characteristics	First stage	Second stage	*p*-value^a^^,^ b
n	1999	1496	
Age (years)	37.54±11.10	37.31±10.80	0.54
Smoking, n (%)			0.66
Yes	1015(50.8)	771(51.5)	
No	984(49.2)	725(48.5)	
Alcohol drinking, n (%)			0.02
Yes	1704(85.5%)	1165(82.6%)	
No	288(14.5%)	246(17.4%)	
Body mass index (kg. m−2)	23.31±3.44	23.46±3.34	0.18

a *T*-test was used to compare means of the continuous variables between the first and the second stages.

b *X* ^2^-test was used to compare the differences for categorical variables.

**Figure 1:**
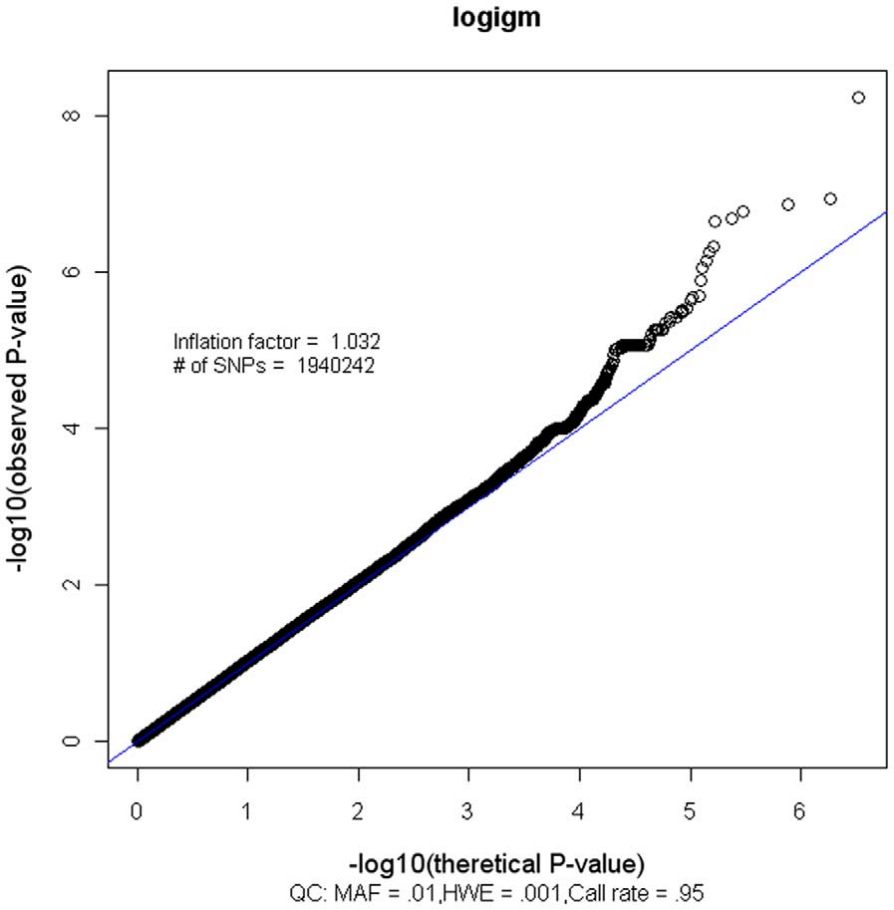
Quantile–quantile Plots.

The quantile-quantile (Q-Q) plot of adjusted *p*-values indicates no systematic bias, with an inflation factor of 1.03 (Figure 1). When the top two Eigens were added to other covariates, the inflation factor remained the same, which indicates no population substructure was observed in our study population.

**Figure 2:**
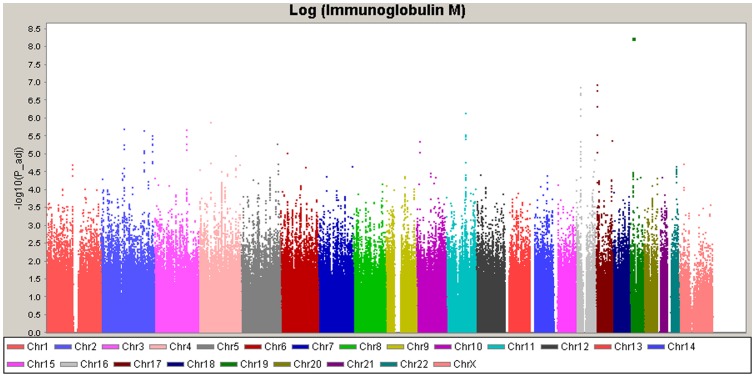
Manhattan plot of genome-wide association analyses for IgM level. X-axis shows chromosomal positions. Y-axis shows −log10 p-values from the linear regression.

The GWAS results were presented in the manhattan plot (Figure 2). In the first stage, we totally identified four loci on two chromosomes that reached a *P*–value of 5.0×10^−7^. After testing the independence of the associated single nucleotide polymorphisms (SNPs) at each of the four loci using the multiple regression analysis, one SNP per region remained to be followed in the second stage (rs11074583, rs11078697, rs11552708, rs11653545). They are located in *PRKCB*, *SENP3*, *TNFSF13*, and *FXR2*, respectively.

In the second stage, only one SNP (rs11552708) was confirmed at *P*-value cutoff of 1.34×10^−3^(adjusting for four tests). When the data from the two stages were combined, the SNP rs11552708 reached a genome-wide significant level of 5.0×10^−8^, with *P*-value of 4.22×10^−9^ (Table 2). SNP rs11552708 was the non-synonymous located in *TNFSF13* gene, which was previously reported to be associated with SLE in several populations [15], [16]. SNP rs11552708 at *TNFSF13* had a significant association with serum IgM levels in our study. And APRIL, which encoded by *TNFSF13*, was reported to have the ability to enhance cell survival and induce the proliferation of B cells as well as stimulating their antibody secretion [15]–[17].

**Table 2:** SNPs associated with levels of IgM.

	Region	position^a^	Genes	Alleles	First stage(N = 1999)						Second stage(N = 1496)					Combined P-values^b^
					Allele(m)	Freq(m)	Mean			P-values^b^	Freq(m)	Mean			P-values^b^	
							mm	mM	MM			mm	mM	MM		
rs11074583	16p11.2	23774414	*PRKCB*	G/A	A	0.6641	1.042	1.1993	1.267	5.67E−08	0.3242	1.16	1.20	1.17	0.50	4.08E−05
rs11078697	17p13	7409953	*SENP3*	T/C	C	0.83	1.039	1.1239	1.254	7.25E−08	0.1596	1.13	1.17	1.19	0.22	4.17E−07
rs11552708	17p13.1	7403279	*TNFSF13*	A/G	G	0.7573	1.034	1.1657	1.26	5.00E−07	0.226	1.08	1.15	1.21	1.34E−3	4.22E−09
rs11653545	17p13.1	7452799	*FXR2*	A/G	G	0.8285	1.043	1.1252	1.253	1.12E−07	0.1603	1.08	1.17	1.19	0.12	2.36E−07

IgM levels were log-transformed and the values presented were back-transformed.

m, minor allele; M, major allele.

We further investigated potential interactions between environmental and genetic factors. And none of the identified SNPs showed statistically significant interactions with smoking or alcohol consumption (all *P*>0.05).

## Discussion

In this two-stage GWAS of 3495 men, we observed that rs11552708 at *TNFSF13* was significantly associated with serum IgM levels, which was consistent with the recent GWAS by Osman et al [18] in Japan. They found 4 SNPs located in 3 loci, including rs11552708 at *TNFSF13* significantly associated with serum IgM levels. Among the 4 SNPs that were implicated in Japanese, 2 were significant at a nominal P-value of 0.05 (Table S2). The SNPs showing no evidence of association in this study, although having previously been established in Japanese, have a statistical power ranging from 5–49%, implying an insufficient sample size for these markers to detect association for IgM. Meanwhile, except for rs3803800, the minor allele frequencies are lower in the Chinese population than that in the Japanese population. Therefore, a larger population is needed to draw a more convincing conclusive about the loci that were not replicated at P of 5E−07, due to the relatively small effect sizes (<0.08). In addition, these discrepancies may be partly caused by differences of the LD blocks in which the SNPs reside between the Chinese and the Japanese.

Rs11552708 was located on chromosome 17p13.1 (Figure S3). It is a non-synoymous polymorphism of the *TNFSF13* gene, codon 67 in exon 1, which leads amino acid substitution in APRIL protein. At amino acid residue 67, the first nucleotide G of the codon GGG for Gly was replaced by A, which resulted in an amino acid change from Gly to Arg (G67R). APRIL (A proliferation-inducing ligand, also known as TNFSF13A/TALL-2) is coded by *TNFSF13*. It is a type II membrane protein of 250 amino acids and its extracellular domain is cleaved at the RKRR motif of amino acid 101–104 by a furin convertase and then secreted-[19]. Being a member of the TNF family-[20], APRIL is believed to have a close relationship with BAFF (B-cell activation factor, also called BLyS, THANK, TALL-1, and zTNF4) [21]. Similar with BAFF, APRIL primarily expresses on monocytes/macrophages and binds to both receptors–TACI (transmembrane activator and calcium modulator and cyclophilin ligand) and BCMA (B cell maturation antigen)–and helps in the co-stimulation of primary T and B cells [15], [22]. Both APRIL and BAFF enhance cell survival and induce the proliferation of B cells as well as stimulate their antibody secretion [15]–[17].

APRIL has been previously confirmed to have the ability to affect antibody production [23], and several studies [15], [21], [24]–[26] including human and murine have identified the effect of APRIL on IgM level. Transgenic mice expressing APRIL in T cells (APRIL Tg mice) showed increased levels (approximately twofold) of IgM anti-virus antibodies compared with control mice [21], [24], and mouse APRIL protein are 82% identical with human in the COOH-terminal part of the extracellular domain [15]. In human, APRIL binds to BCMA and TACI, and competes with BLyS for receptor binding. Thus, APRIL–BLyS and BCMA-TACI form a two-ligand and two-receptor pathway involved in stimulation of B and T cell function. Those interactions induce class switch recombination in human and murine B cells [27], [28]. As a result, the production of IgM was increased by B cell's active [15], [25]. On the other hand, TACI-Fc, which blocks the interaction of APRIL and cellular TACI, can inhibit antibody production [25], [26]. Treatment of mice with a TACI–Fc fusion protein after antigenic challenge diminished IgM production [25]. This may reveal the relationship between APRIL and IgM from another side.

APRIL was originally described to stimulate growth of tumor cells in vitro and in vivo [29], which have the unique capacity to stimulate the growth of transformed tumor cell lines. APRIL is over-expressed in tumor cell lines and some primary tumors, especially lymphomas, and stimulates tumor cell growth [20]. Besides, previous studies have reported the function of stimulates B- and T-cell proliferation, triggers humoral immune responses, activates nuclear factor–B (NF-B), and induces cell death. However, the physiological significance of APRIL has not been fully elucidated [30], [31]. Common variable immunodeficiency (CVID), a syndrome diagnosed on the basis of an impaired ability to produce specific antibodies, markedly reduced serum levels of IgG, IgA and IgM (frequently) and exclusion of other causes for antibody deficiency [31], was also found to relate with APRIL [32], [33].

Moreover, there are also GWAS reporting the impact of *TNFSF13* gene on systemic lupus erythematosus (SLE) [34], [35] and Celiac Disease susceptibility [36]. As to SLE, the related locus is rs11552708, similar with our study, and the result has also been replicated in different populations (Japanese, European-American, African-American and Hispanic) [34], [35]. Interestingly, SLE is an autoimmune disease related to apoptosis [37] and associate with IgM [37], [38]. Therefore, those GWAS may also give us suggestion about relationship between rs1155708 and IgM level. And our study provided GWAS evidence to support the relationship between *TNFSF13* rs11552708 polymorphism and IgM level in Chinese male population.

Our study firstly reported that rs11552708 at *TNFSF13* was related to serum IgM level in a healthy Chinese male population using a two-stage GWAS. However, this study only focused on male participants, and can't offer the data on female participants about the relationship of rs11552708 at *TNFSF13* with serum IgM levels.

In summary, we performed a two-stage GWAS in a Chinese male population to explore the genetic influence on serum IgM level. Our study observed the *TNFSF13* rs11552708 polymorphism was significantly associated with serum IgM, which suggested that *TNFSF13* may be a susceptibility gene affecting serum IgM levels in Chinese male population. Further studies on the association of *TNFSF13* gene with IgM in other populations are warranted.

## Supporting Information

Figure S1 A histogram of the log transformed IgM for stage one.
(DOC)

Figure S2 A histogram of the log transformed IgM for stage two.
(DOC)

Figure S3 Regional plots for the associations of the SNPs at chr17 with serum IgM levels in the first stage of GWAS. SNPs plotted with their −log10 (P-values) in the GWAS based on their physical chromosomal positions. Genotyped SNPs are indicated as circles, while imputed SNPs are indicated as quadrangles. The color scheme indicated the linkage disequilibrium displayed as r2 values between all SNPs and the top-ranked SNP (in purple color). The blue lines represent the recombination rates estimated based on HapMap Phase II database. The plots were drawn using Locus Zoom software.
(DOC)

Table S1 The influence of smoking and alcohol drinking on serum IgM level.
(DOCX)

Table S2 Association results of discovery stage for established IgM loci.
(DOCX)
