# Supplementary figures and images for: Genome-Wide Scan Identifies Variant in TNFSF13 Associated with Serum IgM in a Healthy Chinese Male Population
Source: PLoS One. 2012 Oct 31;7(10):e47990. doi: 10.1371/journal.pone.0047990 (PMC3485370; doi:10.1371/journal.pone.0047990)

**Supplementary Figure S1:** A histogram of the log transformed IgM for stage one


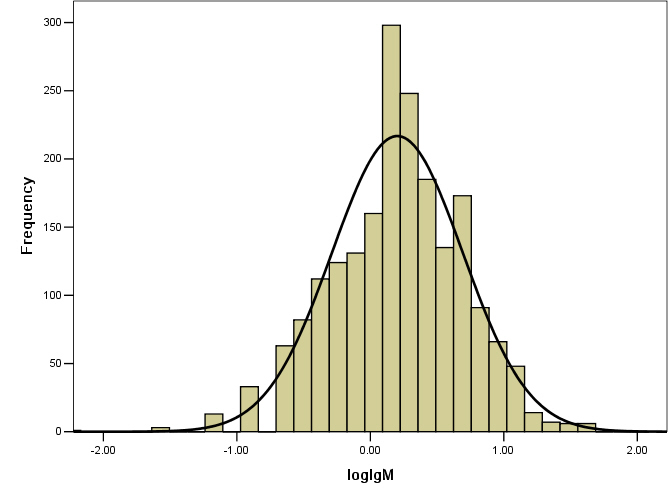

Supplement: Figure S1 — A histogram of the log transformed IgM for stage one. (DOC) [file pone.0047990.s001.doc]

**Supplementary Figure S2:** A histogram of the log transformed IgM for stage two.


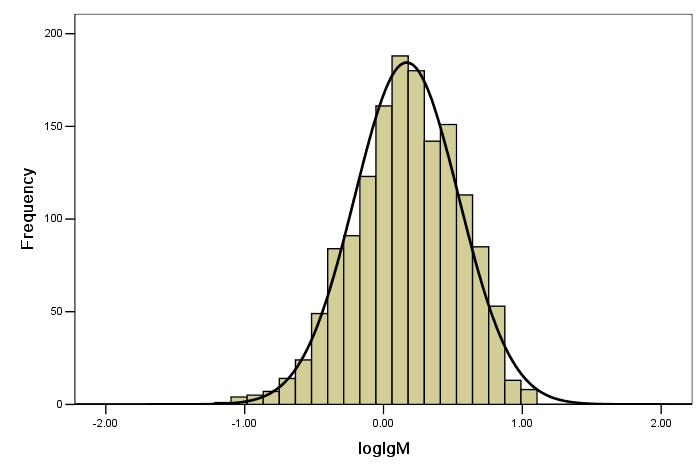

Supplement: Figure S2 — A histogram of the log transformed IgM for stage two. (DOC) [file pone.0047990.s002.doc]
