# Supplementary material for: Genome-Wide Scan Identifies Variant in TNFSF13 Associated with Serum IgM in a Healthy Chinese Male Population
Source: PLoS One. 2012 Oct 31;7(10):e47990. doi: 10.1371/journal.pone.0047990 (PMC3485370; doi:10.1371/journal.pone.0047990)

**Supplementary Figure S3: Regional plots for the associations of the SNPs at chr17 with serum IgM levels in the first stage of GWAS.** SNPs plotted with their –log_10_ (P-values) in the GWAS based on their physical chromosomal positions. Genotyped SNPs are indicated as circles, while imputed SNPs are indicated as quadrangles. The color scheme indicated the linkage disequilibrium displayed as r^2^ values between all SNPs and the top-ranked SNP (in purple color). The blue lines represent the recombination rates estimated based on HapMap Phase II database. The plots were drawn using Locus Zoom software.


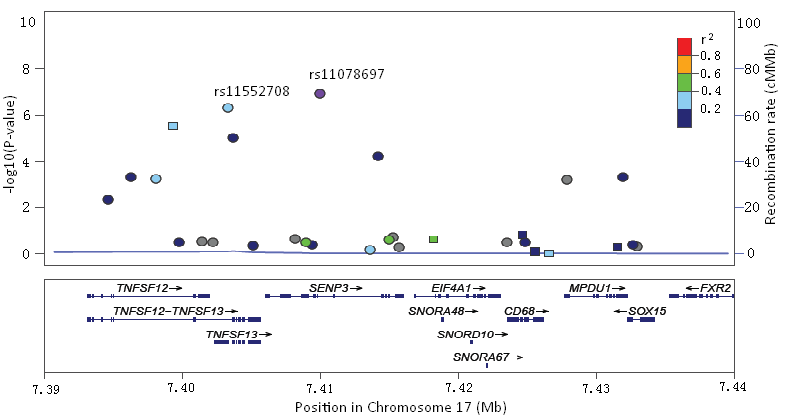

Supplement: Figure S3 — Regional plots for the associations of the SNPs at chr17 with serum IgM levels in the first stage of GWAS. SNPs plotted with their −log10 (P-values) in the GWAS based on their physical chromosomal positions. Genotyped SNPs are indicated as circles, while imputed SNPs are indicated as quadrangles. The color scheme indicated the linkage disequilibrium displayed as r2 values between all SNPs and the top-ranked SNP (in purple color). The blue lines represent the recombination rates estimated based on HapMap Phase II database. The plots were drawn using Locus Zoom software. (DOC) [file pone.0047990.s003.doc]
