# Supplementary material for: Genome-Wide Scan Identifies Variant in TNFSF13 Associated with Serum IgM in a Healthy Chinese Male Population
Source: PLoS One. 2012 Oct 31;7(10):e47990. doi: 10.1371/journal.pone.0047990 (PMC3485370; doi:10.1371/journal.pone.0047990)
Supplement: Table S1 — The influence of smoking and alcohol drinking on serum IgM level. (DOCX) [file pone.0047990.s004.docx]

Supplementary table S1: The influence of smoking and alcohol drinking on serum IgM level

| Characteristic | First stage | | |  | Second stage | | |
| --- | --- | --- | --- | --- | --- | --- | --- |
|  | n | IgM(g/l) | *P ^b^* |  | n | IgM(g/l) | *P ^b^* |
| Smoking |  |  |  |  |  |  |  |
| Yes | 1709 | 1.36±0.66 | 0.001 |  | 1233 | 1.26±0.46 | 0.046 |
| No | 290 | 1.47±0.76 |  |  | 261 | 1.30±0.47 |  |
| Alcohol drinking |  |  |  |  |  |  |  |
| Yes | 1015 | 1.32±0.69 | 0.013 |  | 771 | 1.24±0.46 | 0.129 |
| No | 984 | 1.43±0.67 |  |  | 712 | 1.29±0.46 |  |

Values are shown as mean±s.d; IgM level was log-transformed and the values presented were back-transformed. ^b^T-test was used to compare the means between the sub-group of smoking or drinking.
