# Supplementary material for: Genome-Wide Scan Identifies Variant in TNFSF13 Associated with Serum IgM in a Healthy Chinese Male Population
Source: PLoS One. 2012 Oct 31;7(10):e47990. doi: 10.1371/journal.pone.0047990 (PMC3485370; doi:10.1371/journal.pone.0047990)
Supplement: Table S2 — Association results of discovery stage for established IgM loci. (DOCX) [file pone.0047990.s005.docx]

Supplementary table S2: Association results of discovery stage for established IgM loci

|  |  | Our study^＄^(Chinese, discovery stage) | | | | | | | Japanese﹡(discovery stage) | | |
| --- | --- | --- | --- | --- | --- | --- | --- | --- | --- | --- | --- |
| Candidate genes | SNP | CHR | MAF(allele) | Beta(se)^§^ | P-value | power |  | MAF(allele) | | Beta(se)^§^ | P-value |
| *TNFRSF13B* | rs4985726 | 17 | NA | NA | NA | NA |  | 0.375(G) | | 0.148(0.015) | 2.40E-22 |
| *TNFSF13* | rs3803800 | 17 | 0.403(A) | 0.068(0.015) | 9.28E-06 | 0.27 |  | 0.311(A) | | 0.108(0.015) | 1.80E-12 |
| *TNFSF13* | rs11552708 | 17 | 0.243(A) | -0.088(0.018) | 4.82E-07 | 0.49 |  | 0.401(A) | | -0.084(0.015) | 7.00E-09 |
| *ANXA3* | rs10007186 | 4 | 0.209(C) | -0.002(0.018) | 8.42E-01 | 0.05 |  | 0.307(C) | | 0.095(0.016) | 3.30E-09 |

^＄^sample size, 1999; ﹡sample size, ; NA, data not available; The power was estimated given the discovery stage sample size, discovery stage effect size, Chinese population mean variable and the Chinese minor allele frequency for α=5E-07.
